# Semi-supervised clustering of fractionated electrograms for electroanatomical atrial mapping

**DOI:** 10.1186/s12938-016-0154-5

**Published:** 2016-04-26

**Authors:** Andres Orozco-Duque, John Bustamante, German Castellanos-Dominguez

**Affiliations:** Bioengineering Center, Universidad Pontificia Bolivariana, Medellin, Colombia; GI2B, Instituto Tecnologico Metropolitano, Medellin, Colombia; Signal Processing and Recognition Group, Universidad Nacional de Colombia - sede Manizales, Manizales, Colombia

**Keywords:** Atrial fibrillation, Electrogram-guided ablation, Feature extraction, Spectral clustering

## Abstract

**Background:**

Electrogram-guided ablation procedures have been proposed as an alternative strategy consisting of either mapping and ablating focal sources or targeting complex fractionated electrograms in atrial fibrillation (AF). However, the incomplete understanding of the mechanism of AF makes difficult the decision of detecting the target sites. To date, feature extraction from electrograms is carried out mostly based on the time-domain morphology analysis and non-linear features. However, their combination has been reported to achieve better performance. Besides, most of the inferring approaches applied for identifying the levels of fractionation are supervised, which lack of an objective description of fractionation. This aspect complicates their application on EGM-guided ablation procedures.

**Methods:**

This work proposes a semi-supervised clustering method of four levels of fractionation. In particular, we make use of the spectral clustering that groups a set of widely used features extracted from atrial electrograms. We also introduce a new atrial-deflection-based feature to quantify the fractionated activity. Further, based on the sequential forward selection, we find the optimal subset that provides the highest performance in terms of the cluster validation. The method is tested on external validation of a labeled database. The generalization ability of the proposed training approach is tested to aid semi-supervised learning on unlabeled dataset associated with anatomical information recorded from three patients.

**Results:**

A joint set of four extracted features, based on two time-domain morphology analysis and two non-linear dynamics, are selected. To discriminate between four considered levels of fractionation, validation on a labeled database performs a suitable accuracy (77.6 %). Results show a congruence value of internal validation index among tested patients that is enough to reconstruct the patterns over the atria to located critical sites with the benefit of avoiding previous manual classification of AF types.

**Conclusions:**

To the best knowledge of the authors, this is the first work reporting semi-supervised clustering for distinguishing patterns in fractionated electrograms. The proposed methodology provides high performance for the detection of unknown patterns associated with critical EGM morphologies. Particularly, obtained results of semi-supervised training show the advantage of demanding fewer labeled data and less training time without significantly compromising accuracy. This paper introduces a new method, providing an objective scheme that enables electro-physiologist to recognize the diverse EGM morphologies reliably.

## Background

Atrial Fibrillation (AF) implies that the electrical activity of the atria is highly disorganized, and any coherent mechanical contraction is missed. AF, which is the most common supraventricular arrhythmia, is associated with many cardiac conditions, including an increased risk of thromboembolic events, stroke and heart failure.

Catheter ablation has became an alternative to cure AF, and may avoid side effects of long term pharmacotherapy. Radiofrequency ablation treatment is the generation of tissue injuries which block propagation of electrical impulses to prevent the formation and maintenance of fibrillatory conduction. Catheters for radiofrequency ablation are guided inside the heart chambers via cardiac mapping systems [1].

Although electrical disconnection of the pulmonary veins remains the mainstream procedure of catheter ablation, patients with persisten AF demand more extensive ablation [2]. Recent approaches aim at guiding the ablation using electrical signals recorded inside the atria, called electrograms (EGM). These recordings are incorporated into an electroanatomical mapping system to visualize the 3D distribution of the electrical information through the anatomical atrial structure (electroanatomical atrial mapping – EAM). The main goal of EAM is to locate AF sources outside the region of pulmonary veins in cases of persistent AF.

Even though the mechanism of AF remains unclear, some studies have shown that the EGM morphology during AF may be correlated with different conduction patterns, e.g., conduction blocks, slow conduction, a collision of activation waves or reentries [3]. In fact, areas rendering EGM recordings with remarked high-frequency content or chaotic patterns should be associated with AF [4, 5]. Thus, electrogram-guided ablation procedures have emerged as alternative strategy consisting of either mapping and ablating localized reentrant sources driving AF or targeting complex fractionated electrograms (CFAE) [6]. In accordance to [7], CFAE is formally defined as follow: (1) atrial electrograms that have fractionated electrograms composed of two deflections or more, and/or perturbation of the baseline with continuous deflection of a prolonged activation complex over a 10 s recording period; (2) atrial electrograms with a very short cycle length (≤120 ms) over a 10 s recording period. This inexact and wide-sense statement of CFAE makes the decision of selecting the target sites for ablation to be dependable on the expertise of the electrophysiologist, jeopardizing the effectivity of the CFAE ablation [8, 9]. To overcome these limitations, designation of different levels of fractionation (usually, between three and five) have been proposed based on the perturbation of baseline and the presence of continuous deflection [10, 11]. Every one of the fractionation levels and EGM morphologies remains not well described or is differently defined in the literature, making difficult their discrimination even for the electro-physicians. Therefore, there is a need for an objective scheme capable of distinguishing the diverse morphologies of EGM signals.

The extensive number of the feature extraction methods for the CFAE detection falls into the following categories: (i) features based on time-domain morphology analysis, e.g., measures of the cycle length [12], quantification of deflections [11], characterization of baseline and wave similarity measure [13], among others; (ii) based on frequency analysis, e.g., dominant frequency and regularity index [14]; and (iii) based on nonlinear dynamics, such as Shannon entropy [15] and approximate entropy [16]. All these features aim at distinguishing each level of fractionation by building a single map encoding waveform differences of CFAE upon the anatomical structure of the atria [16]. Although most studied features have a simple implementation, they demand tuning of parameters that in practice should be heuristically fixed. Besides, because of the substantial stochastic behaviour of CFAE, the extraction of a unique feature has been proved to be not enough to identify all distinct substrates perpetuating the arrhythmia [17]. To date, feature extraction from complex fractionated electrograms is carried out based on mostly the time-domain morphology analysis and non-linear features instead of handling the entire waveform directly. However, we employ their combination that has been reported to achieve better performance [18].

On the other hand, most of the inferring approaches applied for identifying CFAE levels of fractionation are supervised. Examples are given in [19, 20], where sets of labeled signals must be used during the training process. Nonetheless, supervised learning is limited by the availability of marked CFAE, which in turn faces two restrictions: the lack of a standard for their objective description [17, 21, 22] and the fact that some of the CFAE properties may vary under the influence of different catheters or acquisition settings [23].

In order to overcome the above-described limitations, this work proposes an semi-supervised clustering method of four levels of fractionation. In particular, we use a spectral clustering that groups a set of widely used atrial EGM features extracted from complex fractionated electrograms. We also introduce a new atrial-deflection based feature quantifying the fractionated activity. Further, we select, from the input feature set, the optimal subset that yields the best performance. For purposes of evaluation of the proposed clustering method, we carry out training for two scenarios: (*a*) *External validation* using a labeled database with four different classes of atrial EGM. (*b*) *Internal validation* in a semi supervised fashion that employs the feature set extracted in the external validation, aiming to perform semi-supervised clustering on an unlabeled dataset recorded from three patients. The obtained results indicate that the proposed method is suitable for automatic identification of critical patterns in AF.

**Fig. 1:**
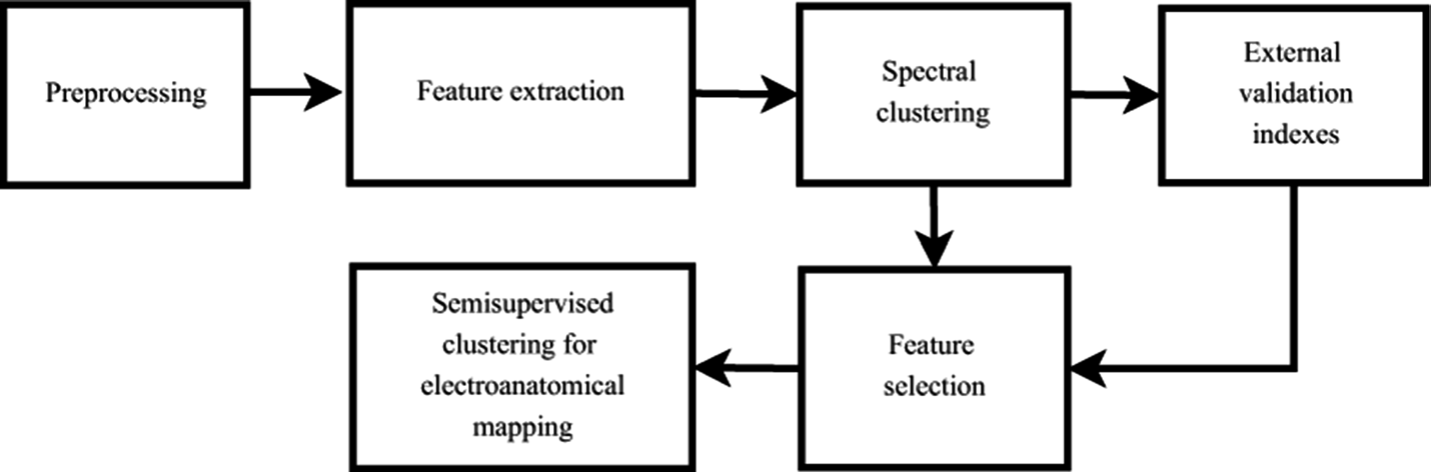
Proposed methodology. Block scheme of the proposed methodology of clustering EGM features to locate critical EGM morphologies in AF

**Fig. 2:**
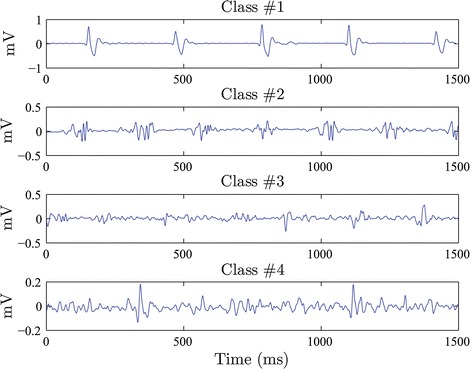
EGM classes. Exemplary of signals from EGM DB1 showing the four considered fractionation levels (class labels): $\#0$, $\#1$, $\#2$, and $\#3$

This work is organized as follows: in "Methods" section methods of feature extraction, spectral clustering, and feature selection are described. "Results of clustering" section carry out the results of experiments using both cases of validation on labeled and unlabeled databases. Lastly, we discuss all obtained results and provide conclusions in "Discussion" and "Conclusions" section, respectively.

## Methods

With the aim at clustering EGM features for identification of ablation target areas, the proposed methodology comprises the following stages (see Fig. 1): (*i*) preprocessing, (*ii*) feature extraction, (*iii*) spectral clustering, (*iv*) feature selection, and (*v*) semi-supervised clustering for electro-anatomical mapping that displays the cluster labels in a color-coded overlaid on the reconstructed 3D atrial geometry of a patient.

### Tested EGM databases

#### Labeled EGM database (**DB1**)

This data collection holds 429 EGM recordings acquired from 11 AF patients, as established and reported in [20]. Intracardiac EGM recordings from a multipolar circular catheter were performed after pulmonary vein isolation with a sampling rate of 1.2 kHz. The database was independently annotated by two electrophysiologists, working at different centers, and with proved experience, according to predefined fractionation classes. Atrial EGM signals were checked visually and were labeled according the following fractionation levels (see Fig. 2): Non-fractionated EGM or level 0 (labeled as $\#0$), mild, intermediate, and high ($\#1$, $\#2$, and $\#3$, respectively). Besides, after visual inspection of the experts, the signals having the following particularities had been also sorted out: (i) signals with low quality with very low voltage, (ii) signals that are superimposed on the ventricular far-field components, (iii) signals remain non-stationary over the whole five-seconds recording.

#### Unlabeled EGM database (**DB2**)

This collection was obtained at the Hamilton General Hospital.^1^ Data were recorded from three patients having definite evidence of AF. The amount of 512 observations was acquired by sequential mapping during spontaneous AF before the circumferential ablation. Namely, 223, 88, is the average time between and 201 signals were recorded from the patients labeled as 1, 2, and 3 respectively. After ablation, all patients restored the sinus rhythm. For EGM acquisition, the circular mapping catheter scheme with 20 poles (2-6-4 mm spacing) was used by means of the EAM system Ensite™NavX™(St. Jude Medical™). The catheter remained stationary during four seconds at each observation point. The data were adquired with a sampling rate of 2034.5 Hz. Besides the electrical data, the information about the anatomical model of the left atrial, acquired by the NavX™, were captured. The vertices and polygons to build the mesh that represent the atrial anatomic were also available. Additionally, the system provided the position of the electrode where every EGM was acquired. These information are used to construc an electro-anatomical map of the atrium for each patient.

### Feature extraction from electrogram morphology analysis

**Fig. 3:**
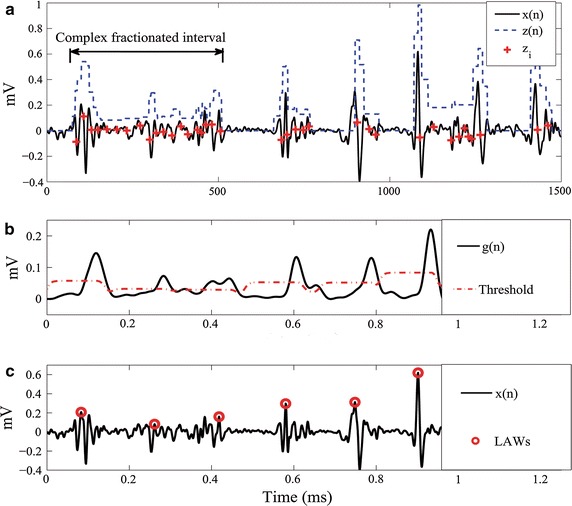
Intraventricular EGM morphology analysis.** a** Detection of atrial deflections.** b** Example of the adaptative threshold and** c** LAW detection

To investigate the anatomic distribution of critical sources in patients with AF, several objective time-based measures are frequently performed, which essentially evaluate the salient organizational properties of the single atrial EGM recordings. Here, the following measures are considered (see Fig. 3):

- *Electrogram deflection time*. Deflections are those perturbations of the EGM baseline having the peak to peak amplitude greater than a given sensibility threshold, $\epsilon _s\in \mathbb {R}^{+}.$ At the same time, the interval between adjacent peaks should last less than a predefined deflection width, $\epsilon _w\in\mathbb {R}^{+}$. Algorithm 1 computes a single vector of time deflections, ${\varvec{\zeta }}\in\mathbb {R}^{n_{d}},$ based on maxima and minima detection computed from the EGM signal.
- *Fractionation interval*. This parameter measures the period between two consecutive deflections (detected within the time range ${\zeta }(j+1) - {\zeta }(j)$) which must be larger than the defined refractory period $\epsilon _r\in\mathbb {R}^{+}$.
- *Complex fractionated interval.* This interval covers uninterrupted electrical activity having consecutive deflection time values shorter than the effective refractory period of the atrial myocardium (70 ms [11]). Besides, all included deflections must exceed 20 % of the amplitude of the highest peak to peak deflection measured over the whole atrial electrogram. Algorithm 2 computes the output vector ${\varvec{z}}\in\mathbb {R}^{N}$ that represent the segments with fractionated electrical activity (see Fig. 3a).
- *Segments of Local Activation Waves (LAW)*. This *p*-samples window holds all events of the local depolarization and is centered on the local atrial activation times (see Fig. 3b, c). For the LAW calculation, each measured atrial electrogram is filtered by a digital, zero-phase, third-order Butterworth filter with passband between 40 and 250 Hz as proposed in [24]. Algorithm 3 performs detection of LAW windows.

Consequently, the following features are extracted from the time-based measurements:

- *Complex fractionated electrogram (CFE) index*, $\xi _1\in\mathbb {R}^{+},$ is the average time between fractionation intervals.
- *Fractionated activity*, $\xi _2\in\mathbb {R}^{+}$ describes the proportion of each EGM signal holding fractionated electrical activity, and is calculated by fixing the time instants when the sign of the envelope changes (i.e., ${\varvec{z}} \ne {0}$). Algorithm 2 computes the envelope ${\varvec{z}}$ of the input signal ${\varvec{x}}$.
- *Variability of segments with fractionated electrical activity*, $\xi _3\in\mathbb {R}^{+}$ is the standard deviation of the width measured for the segments with fractionated electrical activity, ${\varvec{w}}$, (see Algorithm 2).
- *Deflection-LAW ratio*, $\xi _4\in\mathbb {R}^{+},$ is defined by the ratio $\xi _4 = n_d/n_w$, where $n_d$ and $n_w$ are computed from Algorithms 1 and 3, respectively.
- *Similitude index*, $\xi _5\in\mathbb {R}^{+},$ is a wave-morphological resemblance between different local activation waves, quantifying the EGM regularity based on the degree of the LAW repeatability  [13]. This index is defined as follows:
  1 $$\begin{aligned} \xi _5= \frac{2}{(n_w-1)} {\mathbf{\mathbb{E}}} \left\{ {\sum _{j=1}^{n_w}\Theta (\epsilon -\arccos ({\varvec{s}}_i,{\varvec{s}}_j)):\forall i=1,\ldots ,n_w} \right\} \end{aligned}$$
  where ${\Theta }$ is the Heaviside function [25], $\epsilon$ is a threshold adjusted to 0.8,  and ${\varvec{s}}_i$ is the *i*-th detected LAW.
- *Dominant frequency index*, $\xi _6\in\mathbb {R}^{+}.$ This spectral component is inversely proportional to the cycle length. The dominant frequency is computed from the envelope* g* (see Algorithm 3) as the maximum peak of the Fast Fourier Transform power spectrum smoothed by the Hamming window.

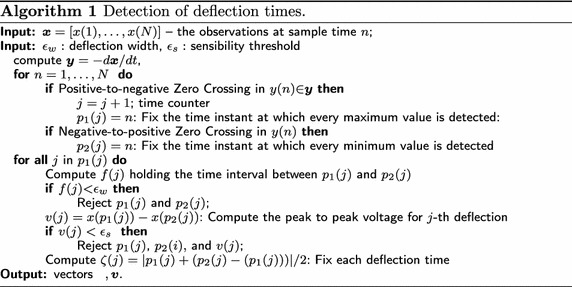

### Non-linear feature extraction from electrograms

Here, based on the non-linear dynamic theory, we also extract the following two non-linear features:

- The approximate entropy, $\xi _{7}\in\mathbb {R}^{+},$ defined by the difference equation:
  2 $$\begin{aligned} \xi _{7}=\Phi ^m(r)-\Phi ^{m-1}(r) \end{aligned}$$
  where $m\in\mathbb {N}$ is the embedded dimension, $r\in\mathbb {R}^{+}$ is a threshold of minimum tolerance, ranging from 0.1 to 0.5 times the standard deviation of the signal. Here, the real-value functional $\Phi ^m(r)\in\mathbb {R}^{+}$ is computed as:
  $$\begin{aligned} \Phi ^m(r)= {\mathbf{\mathbb{E}}} \left\{ {\log {\left( {\mathbf{\mathbb{E}}} \left\{ {\Theta (r-d({\varvec{x}}^{m}_i,{\varvec{x}}^{m}_j)r):\forall j=1,\ldots ,N-m+1}\right\} \right) }: \forall i\ne {j}}\right\} \end{aligned}$$
  where notation ${\mathbf{\mathbb{E}}} \left\{ {\cdot } \right\}$ stands for the expectation operator; $\Theta \in[0,1]$ is the Heaviside function applied to the used measure of similarity between each couple of EGM lagged versions, ${\varvec{x}}^{m}_i$ and ${\varvec{x}}^{m}_j:$
  $$\begin{aligned} d({\varvec{x}}^{m}_i,{\varvec{x}}^{m}_j) = \max _{k= 1,2,\ldots ,m}(|x(i+k-1)-x(j+k-1)|), \end{aligned}$$
  where either lagged vector ${\varvec{x}}^{m}_k=[x(k), \ldots ,x(k-m+1) ]$ (with ${\varvec{x}}^{m}_k\in\mathbb {R}^{m}$) holds the *m* consecutive samples of the original signal, ${\varvec{x}},$ starting at the *i*-th time instant.
- The multifractal *h*-fluctuation index [26], $\xi _8\in\mathbb {R},$ is defined as the power of the second order backward difference of the generalized Hurst exponent $h(q)\in\mathbb {R}$ as follows [26]:
  3 $$\begin{aligned} \xi _8 = \frac{1}{2|q_{\max }|-2}\sum _{q=q_{\min }+2}^{q_{\max }}(h(q)-{2}h(q-1)+h(q-2))^2 \end{aligned}$$
  where $$q\in\mathbb {N}$$ is the order for evaluating the partition function, providing $$q_{\min } < 0, q_{\max } > 0$$ and $$|q_{\min }|=|q_{\max }|;$$$$q_{\min }$$ is the minimum negative order *q*, and $$q_{\max }$$ is the maximum positive order *q* used in the estimation of multi-fractal spectrum through the multi-fractal detrended fluctuation analysis.

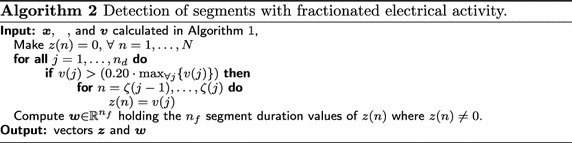

Consequently, we extract $D = 8$ features for identification and localization of critical sources in AF, resulting in the atrial EGM feature point ${\varvec{\xi }}=[\xi _1,\ldots ,\xi _{D}]$ that describes each electrogram.

### EGM feature clustering for identification of ablation target areas

#### Spectral clustering of atrial EGM features

Let ${\varvec{\varXi }}\in\mathbb {R}^{M=D}$ be an input data matrix holding *M* objects and *D* features, where each row $\{{\varvec{\xi }}_i\in\mathbb {R}^{D} : i =1,\dots ,M\}$ denotes one single data point. The goal of clustering is to divide the data into different groups, where samples gathered within the same group are similar to each other. To discover the main topological relationships among data points, spectral clustering-based approaches build from ${\varvec{\varXi }}$ a weighted graph representation $\mathcal {G}\left( {\varvec{\varXi }},{\varvec{K}}\right) ,$ where each object point, ${\varvec{\xi }} \subseteq {\varvec{\varXi }},$ is a vertex or node and ${\varvec{K}}\in\mathbb {R}^{M=M}$ is a similarity (affinity) matrix encoding all associations between graph nodes. In turn, each element of the similarity matrix, $k_{ij} \subseteq {\varvec{K}},$ corresponding to the edge weight between ${\varvec{\xi }}_i$ and ${\varvec{\xi }}_j,$ is commonly defined as follows [27]: $k_{ij}=\mathcal {K}( {\varvec{\xi }}_i,{\varvec{\xi }}_j;\sigma) , \,k_{ij}\in\mathbb {R}^{+},$ where function

$$\begin{aligned} \mathcal {K}({{\varvec{\xi }}}_i,{{\varvec{\xi }}}_j;{\sigma })= \exp \left( -{\Vert {\varvec{\xi }}_i-{\varvec{\xi }}_j\Vert _2^2}/{2\sigma ^2}\right) \end{aligned}$$

is the Gaussian kernel, and $\sigma \in\mathbb {R}^{+}$ is the kernel bandwidth. Notation $\Vert \cdot \Vert _2$ stands for the $L_2$-norm. Although there are many available kernels (like the Laplacian or polynomial ones), the Gaussian function has the advantages of finding Hilbert spaces with universal approximating capability and of being mathematically tractable.

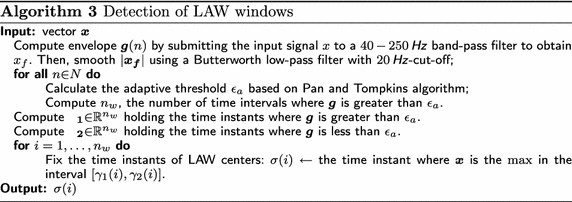



Hence, the clustering task now relies on the conventional graph cut problem that aims at partitioning a set of vertices $\mathcal {V}\in{\varvec{\varXi }}$ into $C\in\mathbb {N}$ disjoint subsets $\mathcal {V}_c,$ so that $\mathcal {V}=\cup _{c=1}^{C} \mathcal {V}_c$ and $\mathcal {V}_{c'} \cap \mathcal {V}_c =\emptyset$, $\forall \; c' \ne c$. Since the graph-cut approaches demand high computational power, relaxation of the clustering optimization problem has been developed based on the spectral graph analysis [28]. So, spectral clustering-based methods decompose the input data ${\varvec{\varXi }}$ into *C* disjoint subsets by using both spectral information and orthogonal transformations of ${\varvec{K}}$. Algorithm 4 describes the well-known solution of the cut problem (termed *NCut*).

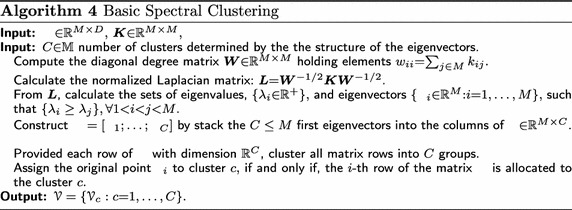



#### Selection of the optimal EGM feature set

Given an input feature matrix ${\varvec{\varXi }}\in\mathbb {R}^{M=D}$, the aim of the feature selection stage is to find the optimal subset ${\varXi }^{*}$ that holds $D' < D$ selected features and provides the highest performance, measured in terms of the cluster validation. For searching ${\varXi }^{*}$, we implemented the Sequential Forward Selection (SFS). At the first iteration, the SFS selects the feature with best performance. In the next iteration, all candidate subsets combining two features (including the one selected before) are evaluated, and so on. This procedure is carried out iteratively by adding all previously selected features and ceases when the following stopping criterion supplies the minimum value:

4 $$\begin{aligned} \mu _{sc}= -(\mu _1-\mu _2),\, \end{aligned}$$

where $\mu _{sc}\in\mathbb {R}[-1,1],$ is the trade-off between the following two indexes of clustering performance: $\mu _1\in\mathbb {R}[0,1]$ is the Adjusted Rand Index that is an external counter checking whether the inferred labels and a set of external labels resemble the same structure [29], and $\mu _2\in\mathbb {R}[0,1]$ is the equivalence mismatch distance that counts all pairs of labels, which have different assignation. Additional explanation about both cluster validation indexes is given in Appendix.

## Results of clustering

For purposes of evaluation of the clustering quality, we carry out training using the selected feature set in two cases: *a*) *External validation* using a labeled database with four different classes of atrial EGM. *b*) *Semi-supervised clustering* that employs a small amount of labeled data, used in the first training case, to aid semi-supervised clustering on unlabeled dataset, associated with anatomical data, performed separately for each patient.

### Parameter setting for feature estimation

In the beginning, each acquired EGM, ${\varvec{x}} \in\mathbb {R}^N$, is firstly submitted to a 30–500 Hz band-pass filter and then passed through a 60 Hz notch filter, being $N=6000$ the signal length. Both procedures are performed by means of the NavX™system.

In order to accomplish the feature extraction stage from the EGM morphology analysis, we detect deflections fixing $\epsilon _w =20$ ms as recommended in [11]. The parameter $\epsilon _s$ is set differently for each database: For DB1, $\epsilon _s=0.01$ of the normalized recording amplitude. For DB2, we fix $\epsilon _s =0.05$ mV since there is just one patient under examination, making unnecessary the normalization of the recordings. Based on the detected set of deflections, the CFE index $\xi _1$ is calculated assuming $\epsilon _r =30$ ms. Besides, the computation of similitude index $\xi _5$ is carried out adjusting $p=90$ ms [13].

For the extraction of the non-linear feature, $\xi _7$, the following parameters are fixed, as suggested in [16]: Embedded dimension $m=3$ and a threshold *r* equal to 0.38 times the standard deviation of the signal. As explained in [16],The optimal value of *r* and *m* is the trade-off between the interclass percentile distance that minimizes the scatter in each class and the interclass minimum-maximum distance that maximizes the distances between the feature measures of the classes. Lastly, calculation of $\xi _8$ is performed from the multifractal detrend fluctuation analysis, where the values $q_{\min }=-5$ and $q_{\max }=5$ are fixed heuristically.

### Clustering-based feature selection

We carry out supervised spectral clustering on DB1 to discriminate between the four levels of fractionation (${C}=4$). As given in [30], we set the kernel parameter $\sigma$ using the tuning method based on the maximization of the transformed data variance as function of the scaling parameter. Further, we complete the feature selection stage that uses all available labels. As shown in Table 1, the most relevant feature is $\xi _2,$ while the selected optimal feature subset is ${\varXi }^{*}=\{\xi _2, \xi _8, \xi _7, \xi _5\}$ which is the one that reaches the best trade-off value of the minimizing cost function $\mu _{sc}.$

**Table 1:** The effect of the choice of features on spectral clustering

Optimal feature set								$\mu _1$	$\mu _2$	$\mu _{sc}$
$\xi _2$								0.459	0.225	−0.234
$\xi _2$	$\xi _8$							0.514	0.197	−0.317
$\xi _2$	$\xi _8$	$\xi _7$						0.491	0.205	−0.286
$\xi _2$	$\xi _8$	$\xi _7$	$\xi _5$					*0.521*	*0.193*	−0.327*
$\xi _2$	$\xi _8$	$\xi _7$	$\xi _5$	$\xi _1$				0.495	0.206	−0.286
$\xi _2$	$\xi _8$	$\xi _7$	$\xi _5$	$\xi _1$	$\xi _4$			0.492	0.235	−0.257
$\xi _2$	$\xi _8$	$\xi _7$	$\xi _5$	$\xi _1$	$\xi _4$	$\xi _3$		0.483	0.235	−0.248
$\xi _2$	$\xi _8$	$\xi _7$	$\xi _5$	$\xi _1$	$\xi _4$	$\xi _3$	$\xi _6$	0.450	0.243	−0.207

Notation ($^\mathbf{* }$) points out on the selected feature subset, ${\varXi }^{*},$ that reaches the lowest value of $\mu _{sc}$

**Fig. 4:**
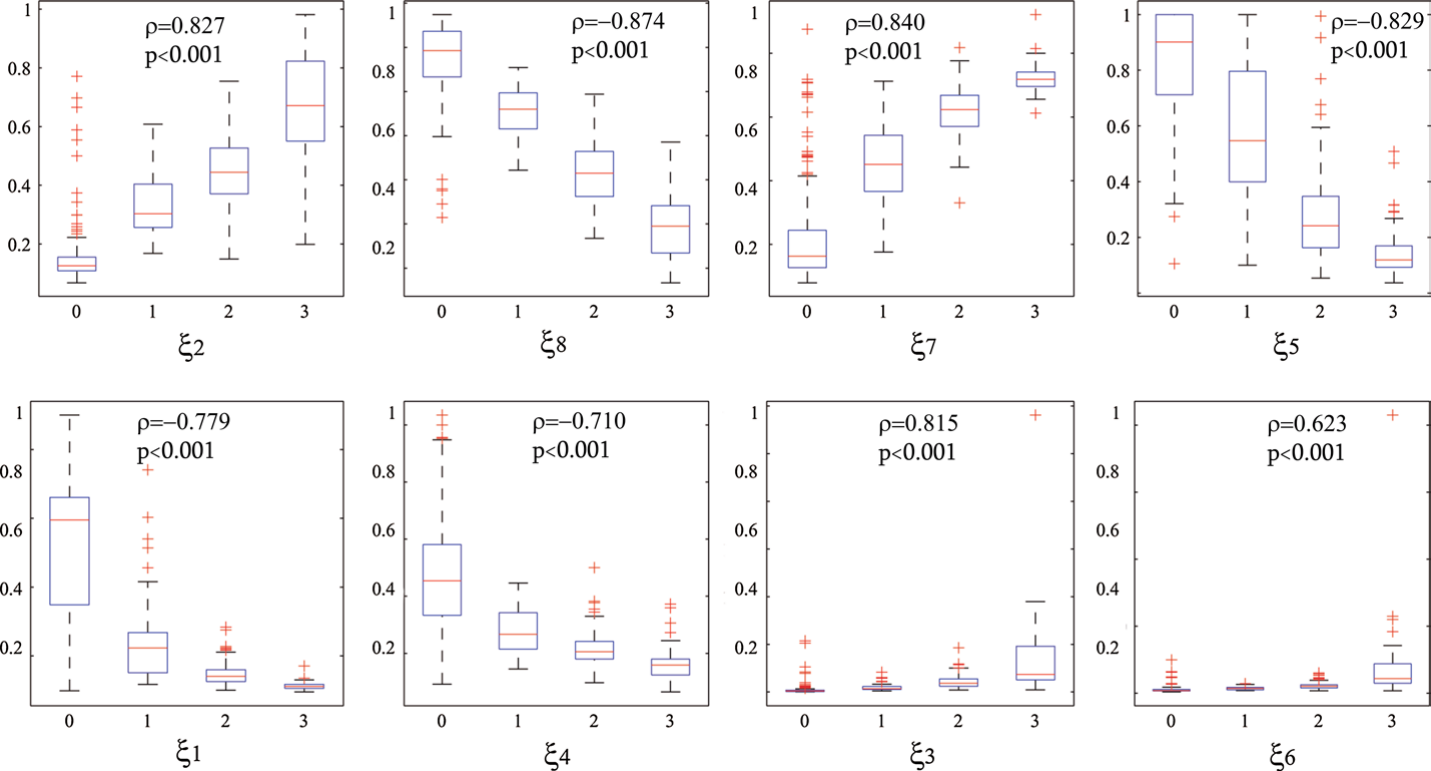
Boxplots of the distribution of features values obtained on the DB1 for all considered levels of fractionation (0, 1, 2 and 3).* Top row*—the selected feature subset ${\varXi }^{*}$;* bottom row*— the rejected feature subset.* Red crosses mark outliers*. All selected features have almost non-overlapping boxplots. This fact illustrates the ability of each feature in separating the classes. Results of Spearman correlation $\rho$ between each feature and the classes of fractionation is shown

Figure 4 displays the boxplot diagrams that include the median values and the interquartile ranges of each feature, calculated for all considered levels of fractionation. In the top row, the boxplot diagrams of the selected feature subset ${\varXi }^{*}$ illustrate the ability of each feature in separating the classes of fractionation levels. All selected features have almost non-overlapping boxplots. This fact favors the distinction of the fractionation levels, since their medians are separated enough from each other. In fact, the results of the carried out Spearman correlation test confirm this assumption. However, a detailed visual inspection of the diagrams shows that the class labeled as $\#0$ (that is, non-fractionated EGM) has the highest number of outliers. By contrast, the class $\#1$ (mild fractionation) holds no outliers at all. In the bottom row, the displayed boxplot diagrams are clearly overlapped, causing that this feature subset to be rejected. Note the poor performance achieved by the features $\xi _3$ (Variability of complex fractionated segments) and $\xi _6$ (dominant frequency index).

### Clustering performance for the external validation

Here, experiments were focused on comparing the clustering results produced by the criterion of feature selection, proposed in Eq. (4), with the ground truth labels provided by DB1. Thus, Spectral clustering was carried out on the selected subset of relevant features, ${\varXi }^{*}.$ For the sake of comparison, we did the same for the complete EGM feature set ${\varXi }$, for the selected morphology-base features, for the selected non-linear features and for the raw-waveform. Table 2 shows the achieved clustering performance measured in terms of sensitivity, specificity, and accuracy for each level of fractionation of DB1. All these performance measures were calculated by direct comparison between the labels provided by an expert and the labels yielded by the spectral clustering technique. Table 2a and b show the computed measures for spectral clustering on subsets ${\varXi }$ and ${\varXi }^{*},$ respectively. As it can be seen, the use of the latter features improves the detection performance remarkably. It is worth noting that the former set ${\varXi }$ includes the CFE index, $\xi _1,$ defection ratio, $\xi _4,$ variability of complex fractionated segments, $\xi _3,$ and dominant frequency index, $\xi _6;$ all these features are related to features extracted from EGM morphology analysis.

**Table 2:** Performed external validating measures of spectral clustering on the labeled ground truth data **DB1**

(a) Performance using ${\varXi }$		
Acc.	Spec.	Sens.
47.55	93.47	84.31
	53.34	76.00
	85.05	11.48
	100.0	1.88

(b) Performance using ${\varXi }^{*}$		
Acc.	Spec.	Sens.
77.62	98.91	71.24
	85.87	78.66
	88.61	84.45
	97.07	75.47

(c) Confusion matrix using ${\varXi }^{*}$				
	$\#0$	$\#1$	$\#2$	$\#3$
$\#0$	113	36	1	3
$\#1$	1	66	8	0
$\#2$	0	18	115	15
$\#3$	0	0	14	39

(d) Accuracy of different sets		
Morphology-based	Non-linear	Raw waveform
69.46 %	70.86 %	36.6 %

**Fig. 5:**
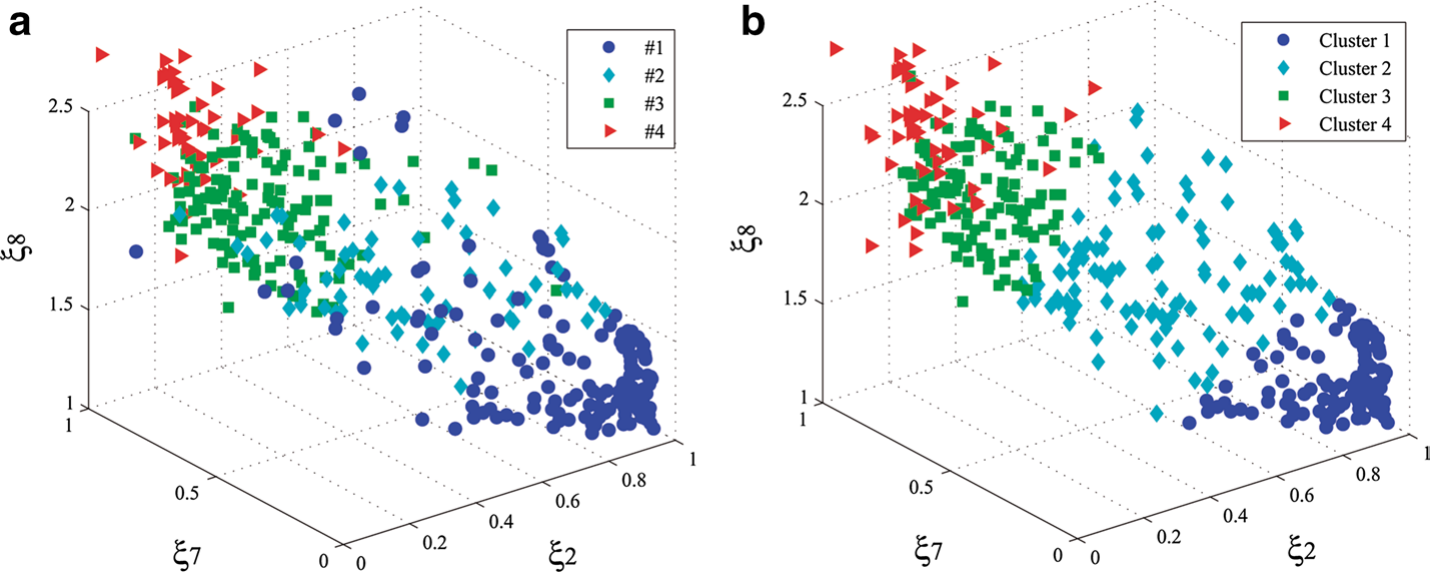
3D scatter plots of the most relevant features extracted from DB1: $\xi _2$, $\xi _7,$ and $\xi _8$.** a** Labeled by experts and** b** Inferred labels by clustering. Both plots resembles the same structure. Clustering tends to locate labels within well-confined class boundaries

**Fig. 6:**
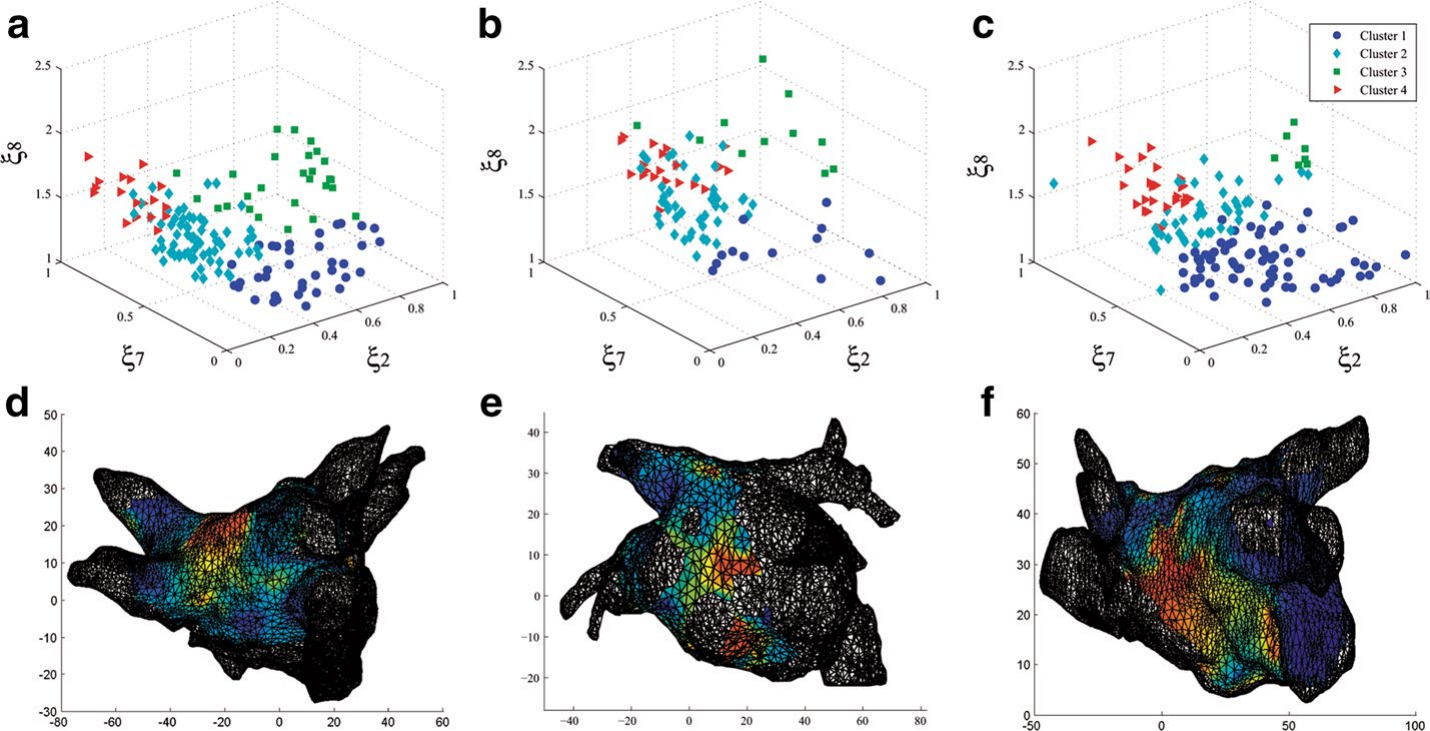
*Clustering scatter plots* and electroanatomical maps for three patients (DB2):** a**–** c** *3D scatter plots* for each patient of the most relevant features: $\xi _2$, $\xi _7,$ and $\xi _8$. Clusters are represented by* colors* and resembles the structure in all three examined patients.** d**–** f**: View of the posterior wall of the left atrium. The clustering results are used to display an electro-anatomical maps for each patient. The* maps* show the distribution of reconstructed EGM patterns over the atrium. Critical sites with hith level of fractionation are* color coded* in* red*

On the other hand, the selected feature set ${\varXi }^{*}$ still supplies low sensitivity for the classes labeled as $\#0$ and $\#3,$ as shown in the corresponding confusion matrix of Table 2(c). For getting a better insight into this issue, Fig. 5 displays 3D scatter plots allowing the visualization of the multivariate features $\xi _2$, $\xi _7,$ and $\xi _8$. As it can be seen in Fig. 5a, which shows the labels assigned by the expert panel, the expert’s markers tend to be more scattered just for the classes $\#0$ and $\#3.$ Apparently, all these spread points are not taken into account by the clustering procedure, as this tends to locate labels within well-confined class boundaries, as shown in Fig. 5b.

### Semi-supervised clustering of unlabeled clinical data

We apply transductive learning to infer the correct labels for the unlabeled samples adquired from the same patient (see DB2), where the cluster assumption holds. Consequently, we assume that unlabeled data tend to form groups clearly separable so that the points of each partition should share one label. The detected EGM classes are handled for visualizing, in a color-coded map, the distribution of the EGM morphologies over the atria in the 3D mesh of the atrium. Thus, the electrophysiologists can locate more accurately the basic EGM classes that have highly fragmented morphologies. To this end, we use just the selected feature set, ${\varXi }^{*},$ that had been inferred by the above-supervised clustering procedure for the labeled data DB1. For the sake of visual inspection, the first row of Fig. 6 displays the estimated 3D scatter plots using the most relevant features ($\xi _2$, $\xi _7,$ and $\xi _8$). As seen in Fig. 6a–c, the location of the clusters resembles the structure in all three examined patients.

To make clear the contribution of this transductive approach, we compare the inferred clusters by quantifying the similarity between partitions achieved for each case of training, supervised and semi-supervised. To this end, the Silhouette Index that ranges within the real-valued interval $[-1,1]$ can be calculated as the ratio of the intercluster cohesion versus to the intracluster separation [31]. Silhouette Index estimates the clustering consistency for each patient, fixing the number of fractionated levels as $C=4.$ The calculated Silhouette Index is 0.471 for patient 1, 0.481 for patient 2 and 0.469 for patient 3, while the same score is 0.57 for DB1, meaning that all carried out partitions tend to be similar in terms of cluster consistency.

The bottom row of Fig. 6 shows three EAM in which all EGM patterns are display over a mesh of the left atrium. The mesh is reconstructed using the anatomical information. EAM allows displaying on color scales the distribution of different EGM classes by their anatomical location at the atrial surface. In this work, the labels assigned by spectral clustering are used for setting the color scale regarding the level of fractionation. The color ranges from the blue that corresponds to non-fractionated signals to the red color standing for the highest level of fractionation. The obtained electroanatomical atrial mapping enables electro-physicians to recognize the location of diverse EGM morphologies on the atrial surface.

## Discussion

In this work, we propose a novel method to construct an semi-supervised-clustering-based electroanatomical map to display the distribution of EGM patterns in the atrial surface. The proposed methodology of training includes the use of a reduced set of features extracted from electrograms, providing a suitable performance. So, our method discriminates four EGM classes and benefits the ablation therapy since it provides an objective scheme that enables electro-physiologist to recognize the diverse EGM morphologies reliably. In accordance with the results obtained in the above section, the following findings are worth mentioning:

- In medical practice, the intracavitary mapping techniques are employed for the ablation in patients suffering from AF. Nevertheless, electrophysiologists must target the critical regions as accurately as possible, aiming to increase the effectiveness of radiofrequency ablation therapy. However, there is an incomplete understanding of the mechanism ruling the AF. Thus, the fractionation levels and EGM morphologies are often vaguely described or differently defined in the professional literature, making very hard their discrimination even for the electro-physicians. This aspect also complicates the automated training. As a result, there a very few available EGM datasets with proper labels. Just, our proposed approach is based on semisupervised clustering when unlabeled data are employed in conjunction with a small amount of labeled data.
- For localization of critical AF drivers in patients with AF, the baseline feature extraction method is grounded on the electrogram morphology analysis. Here, we consider the following five atrial-deflection based features: Complex fractionated electrogram index, fractionated activity, variability, deflection-law ratio, similitude index, and the Dominant frequency index. Two non-linear features are also extracted: Approximate entropy and *h*-fluctuation index. We also carried out feature selection of the optimal subset, yielding the best possible performance of the clustering. Here, the sequential forward selection is implemented, for which we propose a stopping criterion based on the clustering performance. As a result, the following features are selected, ranked by relevance: fractionated activity $\xi _2,$*h*-fluctuation index $\xi _8,$, approximate entropy $\xi _7,$, and similitude index $\xi _5,$. The first feature, fractionated activity index, $\xi _2$, is a time-based measure relating to atrial deflections and describes the proportion of EGM signal holding all segments with fractionated electrical activity. Even though there are other similar indexes reported in literature [10, 32], they require some heuristical thresholds that in practice demand a considerable effort to tune. By contrast, the $\xi _2$ is adjusted according to the effective refractory period of the atrial myocardium, which supplies more reliable physiological information. On the other hand, the following features extracted from electrogram morphology analysis were rejected: the complex fractionated electrogram index $\xi _1$, the defection ratio $\xi _4$, the variability of complex fractionated segments $\xi _3$, and the dominant frequency index $\xi _6$. Furthermore, the relevance of the baseline CFE index $\xi _1$ (termed as CFE-mean in the NavX™system), which has been widely used in some commercial equipments, appears to be very poor, at least in terms of distinguishing among fractionation levels. Clinical studies report that it is unclear whether CFE-index is related with atrial substrates [17]. These results may be explained in the light of the highly non-stationary behavior of the EGM signals, making it difficult to achieve a confident estimation of the time-domain measures performing only the electrogram morphology analysis.
- Even that features extraction from fractionated electrograms is carried out based on mostly the time-domain morphology analysis [11, 33] and non-linear features [15, 16, 34] instead of handling the entire waveform directly, we employ their combination that has been reported to achieve better performance [10, 20]. Our performed training results on the tested database clearly support this statement [see Table 2(d)]: selected morphology-based feature set (69.46 %), selected non-linear set (70.86 %), and selected joint set (77.62 %). For the sake of comparison, we also tested the training using the waveform based input, reaching a very low performance (36.6 %). Obtained results show that the mixture of non-linear and morphology features can more efficiently encode the properties of AF patterns. These findings are consonant with clinical studies that had been carried out for for simulation modeling [15] or animal [5] and human models [35], making the combination of EGM features a promising way to discriminate arrhythmogenic substrates.
- Atrial EGM signals are commonly labeled by three to five fractionation levels due to the influence of the baseline perturbation and continuous deflections [19]. For automating the labeling of ablation target areas, we make use of semi-supervised clustering into four levels of fractionation. Although there are several basic clustering methods, we employ the spectral clustering technique that provides two advantages: performing well with non-Gaussian clusters and totally automated the procedure of parameter settings. Another aspect of consideration is the generalization ability of the used semi-supervised clustering, because it does not make strong assumptions on statistics of the classes. This latter property supplies adequate performance at small patient-specific EGM sets.
- To the best knowledge of the authors, the use of semi-supervised clustering for distinguishing among fractionated levels has not been discussed before. The primary goal of this approach is to make available an automatic training devoted to electroanatomical atrial mapping, avoiding as much as possible the manual classification of AF types and reducing the dependence of prior knowledge about the statistics of the classes. Since manual AF labeling is subjective and time-consuming, it can be achievable for small databases. External validation using a labeled ground truth database with four different levels of fractionation achieved an accuracy of 77.6 %. This performance is comparable to the one (80.65 %) produced by the alternative supervised approach using a fuzzy decision tree in [20]. However, the supervised methods of classification, trained with short training datasets, tend to be biased due to the subjective labeling of AF types suffers from poorly described patterns and strong assumptions on statistics of the classes. This is an important property in this application due the lack of a standard definition of fractionated EGM. In fact, the generalization ability of the proposed training approach is tested to aid semi-supervised learning on unlabeled dataset recorded from three patients. The relevance of locating EGM patterns is encouraged by several studies pointing out that some particular fractionated morphologies are likely to represent drivers of AF [36]. Moreover, experimentation on isolated animal hearts has shown that the areas with highest fractionated EGM signals coexist in the periphery of the most rapid and less fractionated places [4, 37]. This fact may lead to the localization of AF sources and implies that the localization of different patterns, over the patient atrial surface, can become an adequate diagnostic support tool for locating target sites for ablation.
- The proposed methodology of training is devoted to automatic identification of different patterns in atrial EGM during AF. The commonly used systems to perform ablation (NavX system or Carto system) have a limited number of simultaneous EGM electrodes [11]. This fact implies that the EGM signals are asynchronous, and the reconstruction of action potential propagation around the whole atria is unfeasible. The proposed semi-supervised training allows inferring unknown patterns, which can be correlated with AF critical areas, so that it can improve the performance of the ablation therapy, even if the conventional mapping catheter is employed.
- Although electrical isolation of pulmonary veins is the mainstream ablation procedure for AF, CFAE ablation together with pulmonary vein isolation has attracted attention in reducing the long-term recurrence of AF [38]. Nevertheless, the latter ablation remains a debated issue due to the uncertainty of interpretation about many CFAE morphologies [36]. In this regard, the proposed semi-supervised mapping method can favor the use of EGM-guided ablation due to its ability for locating the distribution of different fractionated EGM patterns over the atrial for persistent AF patients. Therefore, the proposed method could be used in clinical studies to establish a relationship between EGM patterns and drivers that maintain AF, aiming to guide ablation procedures in patients with persistent AF.
- Lastly, we measure the computational complexity of the method in terms of processing time. The feature extraction step lasts 2  s for each signals. Provided a testing set that holds 220 EGM signals (the average amount of signals for a mapping procedure), the spectral clustering lasts 0.56 s, and the mapping construction takes only 0.47 s. This time was calculated using MatLab 2013a in a PC with Windows 8 (64 bits), Core I7 processor and RAM of 6 GB. In total, the proposed training algorithm takes a short period so that the method can be employed for clinical purposes.

## Conclusions

This paper introduces a new method for semi-supervised clustering of fractionated electrograms, providing an objective tool for reliably locating the distribution of different fractionated EGM patterns over the atrial. The obtained electroanatomical atrial mapping enables electrophysiologist to locate the critical EGM patterns as accurately as possible, aiming to increase the effectiveness of radiofrequency ablation therapy for persistent AF patients.

Also, we introduce a new atrial-deflection based feature (termed fractionated activity) that does not demand any heuristical parameter tuning, providing an increased discrimination ability in comparison to the other state-of-the-art features. Furthermore, our carried out feature selection allows coming to the conclusion that some used in practice features (like the CFE index) have questionable effectiveness to localization of critical sources in patients with AF. Also, the use of semi-supervised clustering facilitates the automatic detection of fractionation classes with accuracy comparable to other similar results reported in the literature, avoiding the manual labeling of AF classes that is subjective and very time-consuming.

As the future work, the authors plan to improve the performance of the discussed semi-supervised clustering of features extracted from fractionated electrograms. Besides, a more detailed study should be carried out to discriminate different patterns over the atrial surface to be further associated with the fibrillatory conduction. We also plan to conduct clinical assessment of the effectiveness of the proposed method as a new electro-anatomical mapping tool to guide ablation procedures in AF.

## Appendix: Measures of cluster validation

The Adjusted Rand Index (ARI) is an external counter that checks whether the labels of the used clustering procedure, $\mathcal {V},$ and a set of external labels, $\mathcal {V'},$ resemble the same structure. ARI counts each pair-wise verification affiliating objects to the following subsets: Subset *a*) objects labeled in the same cluster of $\mathcal {V}$ and $\mathcal {V'}$, *b*) objects labeled in the same cluster of $\mathcal {V}$, but in different clusters of $\mathcal {V'}$, *c*) objects labeled in the same cluster of $\mathcal {V'},$ but in different cluster of $\mathcal {V}$; and *d*) objects labeled in the different cluster of both $\mathcal {V}$ and $\mathcal {V'}$. Provided the above subsets, ARI is rated as follows [29]:

$$\begin{aligned} \mu _1 =\frac{\left( {\begin{array}{c}M\\ 2\end{array}}\right) (a+d)-((a+b)(a+c)+(c+d)(b+d))}{\left( {\begin{array}{c}M\\ 2\end{array}}\right) ^2-((a+b)(a+c)+(c+d)(b+d))} \end{aligned}$$

ARI has the lowest expected value zero for independent clusterings and maximum value 1 for identical clusterings. At the same time, we minimize the equivalence mismatch distance, termed Mirkin Index, that counts all disagreements pairs *b* and *c* as follows:

$$\begin{aligned} \mu _2=(b+c)/(a+b+c+d) \end{aligned}$$

## Abbreviations

AF: atrial fibrillation
EGM: electrograms
EAM: electro-anatomical atrial mapping
CFAE: complex fractionated atrial electrogram
LAW: local activation waves
CFE: complex fractionated electrogram
SFS: sequential forward selection
DB1: labeled EGM database
DB2: unlabeled EGM database

## Footnotes

1 http://www.phri.ca/.
